# Nanomedicines: current status and future perspectives in aspect of drug delivery and pharmacokinetics

**DOI:** 10.1007/s40005-017-0370-4

**Published:** 2017-11-28

**Authors:** Young Hee Choi, Hyo-Kyung Han

**Affiliations:** College of Pharmacy and Integrated Research Institute for Drug Development, Dongguk University-Seoul, 32 Dongguk-lo, Ilsandong-gu, Goyang, Gyonggi-do 10326 Republic of Korea

**Keywords:** Nanomedicines, Pharmacokinetics, Delivery, Guidelines

## Abstract

Nanomedicines have evolved into various forms including dendrimers, nanocrystals, emulsions, liposomes, solid lipid nanoparticles, micelles, and polymeric nanoparticles since their first launch in the market. Widely highlighted benefits of nanomedicines over conventional medicines include superior efficacy, safety, physicochemical properties, and pharmacokinetic/pharmacodynamic profiles of pharmaceutical ingredients. Especially, various kinetic characteristics of nanomedicines in body are further influenced by their formulations. This review provides an updated understanding of nanomedicines with respect to delivery and pharmacokinetics. It describes the process and advantages of the nanomedicines approved by FDA and EMA. New FDA and EMA guidelines will also be discussed. Based on the analysis of recent guidelines and approved nanomedicines, key issues in the future development of nanomedicines will be addressed.

## Introduction

To date, various nanomedicines have been developed and commercially applied in clinical and non-clinical areas. Nanomedicines have shown essential characteristics such as efficient transport through fine capillary blood vessels and lymphatic endothelium, longer circulation duration and blood concentration, higher binding capacity to biomolecules (e.g. endogenous compounds including proteins), higher accumulation in target tissues, and reduced inflammatory or immune responses and oxidative stress in tissues. These characteristics differ from those of conventional medicines depending on physiochemical properties (e.g.; particle surface, size and chemical composition) of the nano-formulations (De Jong and Borm 2008; Liu et al. 2011; Onoue et al. 2014). Efforts to develop these characteristics of nanomedicines are likely to make them available for treatment of specific diseases which have not been efficiently controlled using conventional medicines, because nanomedicines allow more specific drug targeting and delivery, greater safety and biocompatibility, faster development of new medicines with wide therapeutic ranges, and/or improvement of in vivo pharmacokinetic properties (Onoue et al. 2014). Many nanomedicines have been used for the purpose of increasing efficacy and reducing adverse reactions (e.g., toxicity) by altering efficacy, safety, physicochemical properties, and pharmacokinetic/pharmacodynamic properties of the original drugs (Dawidczyk et al. 2014). In particular, higher oral bioavailability or longer terminal half-life can be expected in case of orally administered nanomedicines, leading to reduction of administration frequency, dose and toxicity (Charlene et al. 2014; Dawidczyk et al. 2014). Regulation of pharmacokinetic characteristics of nanomedicines can results in significant advances in their utilization. Considerations of pharmacokinetic characteristics of nanomedicines and formulability for development purposes, direction and status of their development, and evaluation systems are thought to have important implications for effective development and use of more effective and safe nanomedicines. Therefore, we will present examples of effective go/stop evaluation stages through a review of pharmacokinetic characteristics and delivery of nanomedicines, and the status and processes of nanomedicine evaluation by global regulatory agencies through comparative analysis.

### Delivery and pharmacokinetics of nanomedicines

Changes in pharmacokinetic characteristics of nanomedicines are due to changes in pharmacokinetic properties of their active pharmaceutical ingredients (API), which include longer stay in the body and greater distribution to target tissues, possibly increasing their efficacy and alleviating adverse reactions (Onoue et al. 2014). Regulation of efficacy and/or adverse reactions of nanomedicines is affected by alteration of pharmacokinetics such as in vivo absorption, distribution, metabolism and excretion in the body.

Physiochemical properties of nanomedicines depend on their composition and formulation, which ultimately affect their efficacy and toxicity (EMA 2015a; TGA 2016). Control of physiochemical properties (e.g. composition or formulation) of nanomedicines and adjustment of the degree of binding between nanomedicines and biomolecules eventually regulate in vivo distribution of nanomedicines (EMA 2015a, b; TGA 2016). For example, it has been reported that the type and amount of binding proteins are significantly reduced when nanomedicines are prepared using PEGylated particles. Further, binding of polysorbate coated particles to ApoE was reported to increase their migration to the brain (EMA 2015a; TGA 2016).

Based on the above concepts connecting and efficacy/toxicity, Table 1 shows targeted delivery methods that can lead to changes in the pharmacokinetics of nanomedicines in the body. Delivery mechanisms of nanomedicines can be divided into intracellular transport, epileptic transport and other types (Table 1). Intercellular transport is regulated and facilitated by intracellularization, transporter-mediated endocytosis, and permeation enhancement through interactions involving particle size and/or cell surface (Francis et al. 2005; Jain and Jain 2008; Petros and DeSimone 2010; Roger et al. 2010). In general, a smaller particle size of nanomedicines increases intercellular transport, which facilitates cell permeation and affects absorption, distribution, and excretion of nanomedicines. In particular, cell internalization by transporter-mediated endocytosis depends on particle size of nanomedicines. When nanomedicine particles are large, opsonization occurs rapidly and their removal from the blood by endothelial macrophages is accelerated. It has been reported that affinity of cell surface transporters to nanomedicines varies depending on the particle size of nanomedicines, and this could also influence rapid removal of large particles from the blood by macrophages. In addition, nanomedicines containing non-charged polymers, surfactants, or polymer coatings which degrade in in vivo due to their hydrophilicity, interact with cell surface receptors or ligands to increase permeability or promote internalization of nanomedicines (Francis et al. 2005; Jain and Jain 2008; Petros and DeSimone 2010; Roger et al. 2010).

**Table 1:** Target delivery characteristics related to pharmacokinetic properties of nanomedicines

Targeting methods	Mechanism	Results
Intercellular transport		
Cell internalization	Caveolar-mediated endocytosis (< 60 nm) Clathrin-mediated endocytosis (< 120 nm)	Difference in intracellular defense mechanism depending on particle size Difference in affinity with cell surface transporter the easier the permeation to affect absorption, distribution and excretion by the smaller the particle size Removal from the blood by macrophages by large particles Increased permeability by changing the interaction with cell surface receptors or ligands by coating with polymers, surfactants
Transporter-mediated endocytosis	Interactions between molecules and nanoparticles by cell surface receptors in in vivo system	
Permeation accelerator	Perturbation of intracellular lipids by fatty acids	
Intracellular transport		
Bioadhesive polymer	Opening reversible tight junction and increase of membrane permeability	Improvement of cytotoxic transport of intrinsic drugs by binding to specific proteins, antibodies and other in vivo polymers Anti-cancer drugs: Minimizing cytotoxicity in normal cells by reducing the anticancer effect of the site where the drug does not reach the tight junction and transferring it to the normal cells Reducing the elimination in lungs during inhalation
Chelator	Opening reversible tight junction and increase of membrane permeability	
Others		
EPR effect	Accumulation in tumor cells	Increased anticancer efficacy through increased permeability to cancerous tissue and prolongation of retention time (ie, accumulation)
Conjugation with antibody, protein, peptide, polysaccharide	Selective delivery to target tissues	Control of delivery to the target using receptor/ligand or physiologic specific days on the surface of the target cell enhances drug efficacy/reduction of adverse reactions
Coated with unhygienic hydrophilic material	Improved stability and transport to mucus, prevention of opsonization	Reduction of macrophage-induced or mucosal instability such that drugs stay in the body for a long time to increase drug efficacy/reduce harmful reactions
Control of particle size to avoid removal by mucilage cilia	Retention extension in lung tissue	Degradation in lung mucosa or alleviation of macrophage action

In addition, nanomedicines improve intracellular transport of active pharmaceutical ingredients through binding involving bioadhesive polymers or chelates (Table 1) (Bur et al. 2009; Des Rieux et al. 2006; Devalapally et al. 2007; Francis et al. 2005; Jain and Jain 2008; Mori et al. 2004; Roger et al. 2010). Increased intracellular trafficking of active pharmaceutical ingredients coupled to specific proteins, antibodies, and others in polymers in vivo occurs due to opening of tight junctions and/or increased membrane permeability. In particular, introduction of such a feature in anti-cancer agents can improve the effect of chemotherapy, including targeting brain tumors which are inaccessible to drugs bound by tight junctions, increasing tumor cell targeting, and reducing normal cell targeting. Cytotoxicity against normal cells can be minimized and anti-cancer efficacy achieved using such a nanomedicine strategy. Reduction of nanomedicine elimination in lungs during inhalation leads to increased due to reduced degradation and removal by lung mucosa or macrophages, resulting in increased drug retention time and movement of drug to the target.

Using the enhanced permeability and retention (EPR) effect, it is possible to increase anti-cancer efficacy through increasing tumor permeation and retention time. The EPR effect also makes it possible to selectively deliver nanomedicines to target tissue via conjugation to an antibody, protein, peptide, or polysaccharide, which can be used to modify delivery of nanomedicines to target tissues using receptor/ligand interactions or other physiologically specific target cell interactions, modulating drug efficacy or adverse reactions. Nanomedicines coated with hydrophilic material have improved stability, and their opsonization or accumulation in mucus is prevented. By inhibiting macrophage-induced or mucosal instability, nanomedicines can be retained in vivo, e.g., in lung tissue for prolonged periods of time through particle size, control and avoiding removal by mucus ciliates, which could lead to degradation or macroscopic effects in lung mucosa (Bur et al. 2009). Therefore, a variety of formulations have been developed to use delivery mechanisms which can control pharmacokinetics and pharmacodynamics of nanomedicines.

### Classification and pharmacokinetic properties of nanomedicines

Nanomedicines exhibit a range of in vivo kinetic characteristics depending on their formulations. In this context, disadvantages and advantages of each type of formulation commonly used in nanomedicines (Devalapally et al. 2007) are summarized, and pharmacokinetic properties of various nanomedicines formulations are shown in Tables 2 and 3.

**Table 2:** Classification of nanomedicines considering pharmacokinetic properties

Formulations		Pharmacokinetic properties		Others
		Advantages	Disadvantages	
Dendrimers	Polysine Poly(amidoamine) PEGylated polylysine Lactoferrin-conjugated	High permeability Release control Drug-selective delivery Improved solubility	Limit of administration routes	Low immunogenicity Blood toxicity
Engineered nanoparticles	Nanocrystal SoluMatrix fine particle Nanosized amorphous	Improved systemic exposure Increased retention time in mucus Various routes of administration	Insufficient persistent emission	Gastric mucosal irritation relief of NSAIDs Toxicity by higher Cmax
Lipid nanosystems	Emulsion Liposome Solid lipid nanoparticle Lectin-modified solid lipid	Degradation or metabolism of formulated materials Improved systemic exposure Drug-selective delivery Accumulation in tumor cells	Quick removal by RES uptake Limit of administration routes	Low toxicity and antigenicity Cytotoxicity due to surfactant
Micelles		High permeability Improved solubility Improved systemic exposure	Insufficient persistent emission	Low immunogenicity Cytotoxicity due to surfactant
Polymeric nanoparticles	Ethyl cellulose/casein PLGA alginate, PLGA PLA-PEG Hydrogel Albumin Chitosan analog	Stable drug release in in vivo Increased retention time of drug	Required initial burst protection Limit of administration routes	Low immunogenicity Required removal of non-degradable polymer

**Table 3:** Specific pharmacokinetic characteristics of drugs based on the classification of nanomedicines

Formulations		API	Techniques	Administration routes	PK properties
Dendrimer		Doxorubicin	Polylysine dendrimer	IV	Increase of systemic exposure, accumulation in tumor cells
		Flurbiprofen	Poly(amidoamine) dendrimer	IV	Increase of distribution and retentions in inflammatory sites
		Methotrexate	PEGylated polylysine dendrimer	IV	Prolongation of systemic exposure
			Lactoferrin-conjugated dendrimer	IV	Accumulation in lungs
		Piroxicam	Poly(amidoamine) dendrimer	IV	Prolongation of systemic exposure
Engineered NPs		Carbendazim	Nanocrystals	PO	Increase of oral F
		Cilostazol	Nanocrystals	PO	Increase of oral F
		Curcumin	Nanocrystals	PO	Increase of oral F
		Danazol	Nanocrystals	PO	Increase of oral F
		Diclofenac	SoluMatrix™ fine particle	PO	Rapid absorption, pain relief
		Fenofibrate	Nanocrystals	PO	Increase of oral F
		Indomethacin	SoluMatrix fine particle	PO	Rapid absorption
		Megestrol acetate	Nanocrystals	PO	Increase of oral F
		Nitrendipine	Nanocrystals	PO	Increase of oral F
		Nobiletin	Nanosized amorphous particles	PO	Increase of oral F, liver protective effect
		Tranilast	Nanocrystals	PO	Increase of oral F, rapid absorption
			Inhalable nanocrystalline powders	Lungs	Increase of anti-inflammatory effect in lungs
		Paclitaxel	Albumin nanoparticles	IV	Tumor targeting
Lipid	Emulsion	Cinnarizine	Self-emulsifying drug delivery	PO	Increase of oral F
		Coenzyme Q10	Solid self-emulsifying delivery	PO	Increase of oral F
		Cyclosporin A	Self-emulsifying drug delivery	PO	Increase of oral F
			Inhalable dry emulsions	Lungs	Increase of anti-inflammatory effect in lungs
		Halofantrine	Self-emulsifying drug delivery	PO	Increase of oral F
		Simvastatin	Self-emulsifying drug delivery	PO	Increase of oral F
	Liposomes	Amikacin	Liposome (Phospholipid/Chol)	IV	Increase of half-life
		Amphotericin B	Liposome (PC/Chol/DSPG)	IV	Increase of systemic exposure, decrease of RES uptake
		Cytarabine/daunorubicin	Liposome (DSPC/DSPG/Chol)	IV	CL reduction
		Doxorubicin	Liposome, PEGylated liposome	IV	Increase of distribution in tumor cells
		O-palmitoyl tilisolol	Liposome (PC/Chol)	IV	Increase of distribution
		Paclitaxel	Liposome (PC/PG)	IV	Prolongation of systemic exposure
		Prednisolone	Liposome (PC/Chol/10% DSPE-PEG2000)	IV	Prolongation and increase of systemic exposure
	Solid lipid NPs	Azidothymidine	Solid lipid NPs	IV	Increase of permeability and retention time in brain
		Clozapine	Solid lipid NPs	IV	Increase of systemic exposure, CL reduction
		Diclofenac Na	Solid-in-oil NPs	Skin	Increase of percutaneous absorption
		Insulin	Lectin-modified solid lipid NPs	PO	Increase of oral F
		Lidocaine	Solid lipid nanoparticles	Skin	Regulation of skin permeability
Micelles		Camptothecin	Block copolymeric micelles	IV	Increase of systemic exposure
		Doxorubicin	Block copolymeric micelles	IV	Increase of systemic exposure, CL reduction
		Paclitaxel	Block copolymeric micelles	IV	Increase of systemic exposure, CL reduction
		Pilocarpine	Block copolymeric micelles	Eyes	Increase of efficacy
		Tranilast	Self-micellizing solid dispersion	PO	Increase of oral F
Polymeric NPs		Celecoxib	Ethyl cellulose/casein NPs	PO	Increase of oral F
		Clotrimazole/econazole	PLGA and alginate NPs	PO	Increase of oral F
		Docetaxel	PLA-PEG NPs	IV	Increase of half-life and anti-cancer effect
		Doxorubicin	PLGA NPs	IV, IP	Increase of half-life, decrease of distribution in heart
		Glucagon	PLGA NPs	Lungs	Increase of half-life, increase of oral F
		Glucagon	PLGA NPs	Lungs	Increase of oral F and half-life
		Insulin	Hydrogel NPs	PO	Increase of oral F
		Rifampicin	PLGA NPs	PO	Increase of oral F
		siRNA	Chitosan analog NPs	PO	Increase of systemic exposure, gene silencing
		VIP derivative	PLGA NPs	Lungs	Anti-inflammatory effect

#### Dendrimers

Dendrimers are characterized by the presence of polysine, poly(amidoamine), PEGylated polylysine, or lactoferrin-conjugated formulations, with high membrane permeability, controlled release ability, selective delivery of active pharmaceutical ingredients, and solubility improvement. There have been reports of limitations in route of administration and immunogenicity, and blood toxicity cases have also been reported (Devalapally et al. 2007; Kawabata et al. 2011; Liu and Fréchet 1999; Mora-Huertas et al. 2010). Applications of dendrimer technology to active pharmaceutical ingredients are exemplified in several reports (Asthana et al. 2005; Barenholz 2012; Chaturvedi et al. 2013; Fanciullino et al. 2013; Feldman et al. 2012; Fetterly and Straubinger 2003; Hanafy et al. 2007; Hrkach et al. 2012; Jia et al. 2003; Jinno et al. 2006; Kaminskas et al. 2011, 2012; Kato et al. 2012; Kawabata et al. 2010; Kurmi et al. 2011; Larsen et al. 2013; Manvelian et al. 2012a, b; Manjunath and Venkateswarlu 2005; Matsumura et al. 2004; Morgen et al. 2012; Onoue et al. 2010a, b, 2011a, b, 2012a, b, 2013a, b; Pandey et al. 2005; Pathak and Nagarsenker 2009; Piao et al. 2008; Pepic´ et al. 2004; Prajapati et al. 2009; Reddy and Murthy 2004; Reddy et al. 2004; Sharma et al. 2004; Strickley 2004; Sylvestre et al. 2011; Teshima et al. 2006; Thomas et al. 2012, 2013; Tomii 2002; Watanabe et al. 2006; Wu and Benet 2005; Xia et al. 2010; Zhang et al. 2006, 2008, 2013). Polylysine dendrimer with doxorubicin, an intravenously administered anti-cancer nanomedicine, results in increased systemic exposure and tumor cell of doxorubicin. Poly(amidoamine) dendrimer with flurbiprofen is an intravenously injectable solution with increased distribution to the site of inflammation and increased in vivo retention time. PEGylated polylysine dendrimer with methotrexate or lactoferrin-conjugated dendrimer with methotrexate are intravenous formulations with prolonged systemic exposure and increased lung accumulation, respectively. Poly(amidoamine) dendrimer with piroxicam with is a formulation with increased systemic exposure.

#### Engineered nanoparticle

Engineered nanoparticles comprise nanocrystals, solumatrix fine particles, or nanosized amorphous particles, which can improve systemic exposure and decrease retention in the mucosal layer. They can be administered via various routes, but result in insufficient sustained release. Examples of engineered nanoparticle application include reducing gastric mucosal irritation due to NSAID nanomedicines, reducing other kinds of toxicity due to high Cmax compared to the original drug (Devalapally et al. 2007; Kawabata et al. 2011; Liu and Fréchet 1999; Mora-Huertas et al. 2010).

Carbendazim, cilostazol, curcumin, danazol, fenofibrate, megestrol acetate, nitrendipine, and tranilast are administered orally by increasing oral bioavailability (F) using nanocrystal formulations. Diclofenac and indomethacin formulations, using SoluMatrix™ fine particle technology, are oral formulations with improved absorption rates and pain relief. Nanosized amorphous particles of Nobilet show reduced hepatotoxicity (i.e., protection of liver function) with oral F. Inhalable nanocrystalline powder of Tranilast is a formulation administered directly to lungs and with improved anti-inflammatory effect. Albumin nanoparticles of paclitaxel improves targeting variability by increasing delivery to cancer cells when intravenously administered (Asthana et al. 2005; Barenholz 2012; Chaturvedi et al. 2013; Fanciullino et al. 2013; Feldman et al. 2012; Fetterly and Straubinger 2003; Hanafy et al. 2007; Hrkach et al. 2012; Jia et al. 2003; Jinno et al. 2006; Kaminskas et al. 2011, 2012; Kato et al. 2012; Kawabata et al. 2010; Kurmi et al. 2011; Larsen et al. 2013; Manvelian et al. 2012a, b; Manjunath and Venkateswarlu 2005; Matsumura et al. 2004; Morgen et al. 2012; Onoue et al. 2010a, b, 2011a, b, 2012a, b, 2013a, b; Pandey et al. 2005; Pathak and Nagarsenker 2009; Piao et al. 2008; Pepic´ et al. 2004; Prajapati et al. 2009; Reddy and Murthy 2004; Reddy et al. 2004; Sharma et al. 2004; Strickley 2004; Sylvestre et al. 2011; Teshima et al. 2006; Thomas et al. 2012, 2013; Tomii 2002; Watanabe et al. 2006; Wu and Benet 2005; Xia et al. 2010; Zhang et al. 2006, 2008, 2013).

#### Lipid nanosystems

Lipid nanosystems including emulsions, liposomes, solid-lipid nanoparticles, and lectin-modified solid lipids can be used to control the degradation and metabolism of the formulation and prolong systemic exposure. In addition, the selective delivery of pharmaceuticals can be improved and the pharmacological effect (e.g. anti-cancer effects in anti-cancer nanomedicines) can be enhanced by the increase of its accumulation in cancer tissues However, their disadvantages include rapid removal due to reticuloendothelial system (RES) uptake, limitation of administration routes, cytotoxicity risk due to low anti-genicity, and surfactant use for formulation (Devalapally et al. 2007; Kawabata et al. 2011; Liu and Fréchet 1999; Mora-Huertas et al. 2010).

Emulsions were formulated to increase oral F in both self-emulsifying and drug delivery systems, and several nanomedicines with emulsion formulations have been clinically used including cinnarizine, coenzyme Q10, cyclosporin A, halofantrine, and simvastatin. Inhalable dry emulsion of cyclosporin A is used to induce an anti-inflammatory effect in the lungs (Devalapally et al. 2007; Kawabata et al. 2011; Liu and Fréchet 1999; Mora-Huertas et al. 2010).

Differences in liposome constituents in liposome formulations have been documented in several reports (Asthana et al. 2005; Barenholz 2012; Chaturvedi et al. 2013; Fanciullino et al. 2013; Feldman et al. 2012; Fetterly and Straubinger 2003; Hanafy et al. 2007; Hrkach et al. 2012; Jia et al. 2003; Jinno et al. 2006; Kaminskas et al. 2011, 2012; Kato et al. 2012; Kawabata et al. 2010; Kurmi et al. 2011; Larsen et al. 2013; Manvelian et al. 2012a, b; Manjunath and Venkateswarlu 2005; Matsumura et al. 2004; Morgen et al. 2012; Onoue et al. 2010a, b, 2011a, b, 2012a, b, 2013a, b; Pandey et al. 2005; Pathak and Nagarsenker 2009; Piao et al. 2008; Pepic´ et al. 2004; Prajapati et al. 2009; Reddy and Murthy 2004; Reddy et al. 2004; Sharma et al. 2004; Strickley 2004; Sylvestre et al. 2011; Teshima et al. 2006; Thomas et al. 2012, 2013; Tomii 2002; Watanabe et al. 2006; Wu and Benet 2005; Xia et al. 2010; Zhang et al. 2006, 2008, 2013). Intravenous injectable solutions of amikacin and O-palmitoyl tilisolol in liposomes (Phospholipid/Chol) have been used for half-life extension, amphotericin B in liposomes (PC/Chol/DSPG) shows decreased systemic exposure and RES uptake, and cytarabine/daunorubicin in liposomes (DSPC/DSPG/Chol) has been used to reduce clearance. Pegylated liposome-treated doxorubicin results in increased distribution of doxotubicin to cancer tissues, and prednisolone in liposomes (PC/PG) or (PC/Chol/10% DSPE-PEG2000) results in prolonged systemic exposure. Solid-lipid nanoparticles of azidothymidine result in increased permeability to the brain, those of clozapine result in increased systemic exposure due to clearance reduction, those of diclofenac developed as a transdermal preparation result in increased transdermal absorption, and those of lidocaine as a transdermal preparation result in longer duration of drug efficacy by regulating skin permeability. A lectin-modified solid-lipid N of insulin shows increased oral F (Asthana et al. 2005; Barenholz 2012; Chaturvedi et al. 2013; Fanciullino et al. 2013; Feldman et al. 2012; Fetterly and Straubinger 2003; Hanafy et al. 2007; Hrkach et al. 2012; Jia et al. 2003; Jinno et al. 2006; Kaminskas et al. 2011, 2012; Kato et al. 2012; Kawabata et al. 2010; Kurmi et al. 2011; Larsen et al. 2013; Manvelian et al. 2012a, b; Manjunath and Venkateswarlu 2005; Matsumura et al. 2004; Morgen et al. 2012; Onoue et al. 2010a, b, 2011a, b, 2012a, b, 2013a, b; Pandey et al. 2005; Pathak and Nagarsenker 2009; Piao et al. 2008; Pepic´ et al. 2004; Prajapati et al. 2009; Reddy and Murthy 2004; Reddy et al. 2004; Sharma et al. 2004; Strickley 2004; Sylvestre et al. 2011; Teshima et al. 2006; Thomas et al. 2012, 2013; Tomii 2002; Watanabe et al. 2006; Wu and Benet 2005; Xia et al. 2010; Zhang et al. 2006, 2008, 2013).

#### Micelles

Micelles have advantages of high membrane permeability, and improved solubility and systemic exposure, but disadvantages of insufficient sustained release and cytotoxicity due to surfactant use (Devalapally et al. 2007; Kawabata et al. 2011; Liu and Fréchet 1999; Mora-Huertas et al. 2010). Block copolymeric micelles reduce clearance and increase systemic exposure of active pharmaceutical ingredients in intravenously administered formulations of camptothecin, doxorubicin, and paclitaxel. Block copolymer micelle allow direct administration to the eyeball increasing its efficacy. Self-micellizing solid dispersion of tranilast result in increased oral F (Asthana et al. 2005; Barenholz 2012; Chaturvedi et al. 2013; Fanciullino et al. 2013; Feldman et al. 2012; Fetterly and Straubinger 2003; Hanafy et al. 2007; Hrkach et al. 2012; Jia et al. 2003; Jinno et al. 2006; Kaminskas et al. 2011, 2012; Kato et al. 2012; Kawabata et al. 2010; Kurmi et al. 2011; Larsen et al. 2013; Manvelian et al. 2012a, b; Manjunath and Venkateswarlu 2005; Matsumura et al. 2004; Morgen et al. 2012; Onoue et al. 2010a, b, 2011a, b, 2012a, b, 2013a, b; Pandey et al. 2005; Pathak and Nagarsenker 2009; Piao et al. 2008; Pepic´ et al. 2004; Prajapati et al. 2009; Reddy and Murthy 2004; Reddy et al. 2004; Sharma et al. 2004; Strickley 2004; Sylvestre et al. 2011; Teshima et al. 2006; Thomas et al. 2012, 2013; Tomii 2002; Watanabe et al. 2006; Wu and Benet 2005; Xia et al. 2010; Zhang et al. 2006, 2008, 2013).

#### Polymeric nanoparticles

Polymeric nanoparticles include ethyl cellulose/casein, PLGA (PLGA and alginate), PLA-PEG, hydrogel, albumin and chitosan analogs with characteristics of relatively stable drug release and prolonged duration of action. However, there are a few cases in which initial rupture is inhibited, or administration routes are limited. In particular, it is necessary to consider factors involved in elimination of non-degradable polymers from the body (Devalapally et al. 2007; Kawabata et al. 2011; Liu and Fréchet 1999; Mora-Huertas et al. 2010).

Polymeric nanoparticles with increased F include ethyl cellulose/casein nanoparticles with celecoxib, PLGA and alginate nanoparticle with clotrimazole/econazole or rifampicin, hydrogel nanoparticle with insulin, and an oral formulation of siRNA using chitosan analog nanoparticles. An docetaxel IV formulation using PLA-PEG nanoparticles showed a prolonged anticancer effect due to increased half-life. IV or IP formulations of LGA nanoparticles with doxorubicin have been reported to show reduced toxicity through prolongation of half-life and reduction of cardiac distribution. Half-life extension and F increase are also reported in the case of PLGA nanoparticles with glucagon (Asthana et al. 2005; Barenholz 2012; Chaturvedi et al. 2013; Fanciullino et al. 2013; Feldman et al. 2012; Fetterly and Straubinger 2003; Hanafy et al. 2007; Hrkach et al. 2012; Jia et al. 2003; Jinno et al. 2006; Kaminskas et al. 2011, 2012; Kato et al. 2012; Kawabata et al. 2010; Kurmi et al. 2011; Larsen et al. 2013; Manvelian et al. 2012a, b; Manjunath and Venkateswarlu 2005; Matsumura et al. 2004; Morgen et al. 2012; Onoue et al. 2010a, b, 2011a, b, 2012a, b, 2013a, b; Pandey et al. 2005; Pathak and Nagarsenker 2009; Piao et al. 2008; Pepic´ et al. 2004; Prajapati et al. 2009; Reddy and Murthy 2004; Reddy et al. 2004; Sharma et al. 2004; Strickley 2004; Sylvestre et al. 2011; Teshima et al. 2006; Thomas et al. 2012, 2013; Tomii 2002; Watanabe et al. 2006; Wu and Benet 2005; Xia et al. 2010; Zhang et al. 2006, 2008, 2013).

### Pharmacokinetic properties of nanomedicines

Pharmacokinetic characteristics of various nanomedicines with different formulations are determined by particle size, shape (chemical structure), and surface chemical characteristics (FDA 2015). Nanomedicines with particle size less than 10 nm are removed by kidneys whereas those with particle size more than 10 nm are sometimes elongated and removed by the liver and/or the mononuclear-phagocyte system (MPS). The aim of regulating particle size in nanomedicines is to increase their retention in target tissues, and to remove them rapidly when distributed to non-target tissues. A protein corona is formed around nanomedicines by non-specific protein adsorption in body, but this is prevented by materials such as polyethylene glycol (PEG) applied on the nano-particle through surface coating. Such protein adsorption induces protein denaturation, which may lead to protein aggregation or phagocytosis due to activated macrophages. Nanoparticle targeting based on chemical properties of nanoparticles and surface coatings comprises active and passive targeting. Passive targeting is defined as non-specific accumulation in disease tissue (usually cancer tissue). This is especially applicable to solid cancers in which targeting results in increased blood vessel and transporter permeations and retention (enhanced permeability and retention, EPR effect) of nanomedicines, and their increased accumulation in tumor tissues. Specific or active targeting is defined as selective transport of nanomedicines containing protein, antibody, or small molecule only to specific tissues and/or specific cells. This may occur via homing to overexpressed cell-surface receptors.

### Pharmacokinetic assessment of nanomedicine by regulatory agencies

As mentioned above, a wide variety of nanomedicine have been developed and approved for use in clinical practice and there are also a number of nanomedicines in clinical trials. As of 2016, 78 nanomedicines were on pharmaceutical markets across the world and 63 nanomedicines were approved as drugs or were in the approval process based on search results from ‘http://www.clinicaltrial.gov’. It would be meaningful to summarize key considerations of the approval authorities and use this knowledge for the development and approval of nanomedicines.

### Food and Drug Administration (FDA)

Nanoscale materials as defined by the US FDA include nanomaterials (materials used in the manufacture of nanomedicine, additives, etc.) and final products (nanomedicine). The particle size of such materials is typically 1–100 nm and such nanomedicines tend to result in increased bioavailability, decreased dose, improved drug efficacy, and decreased toxicity. Improvements in physical properties through effective formulation have led to improved solubility, dissolution rate, oral bioavailability, targeting to specific organs or cells, and/or improved dosage/convenience, leading to dose reduction with less adverse reactions due to the constituent active pharmaceutical ingredients or surfactants (FDA 2015).

#### Status of nanomedicines approved by the FDA

The FDA approved 51 nanomedicines by the year 2016, 40% of which were in clinical trials between 2014 and 2016 (Arnold et al. 2001; Benbrook 2015; Berges and Eligard 2005; Bobo et al. 2016; Desai et al. 2006; Duncan 2014; FDA 2006, 2014, 2015; Foss 2006; Foss et al. 2013; Fuentes et al. 2015; Green et al. 2006; Hann and Prentice 2001; Hu et al. 2012; Ing et al. 2016; James et al. 1994; Johnson et al. 1998; May and Li 2013; Möschwitzer and Müller 2006; Salah et al. 2010; Shegokar and Müller 2010; Taylor and Gercel-Taylor 2008; Ur Rehman et al. 2016; Wang-Gillam et al. 2016) (Table 4). Formulated nanomedicines approved by the FDA can be classified into polymer nanomedicines, micelles, liposomes, antibody-drug conjugates, protein nanoparticles, inorganic nanoparticles, hydrophilic polymers, and nanocrystals. Polymer nanomedicines are the simplest forms of nanomedicines and contain soft materials to increase solubility, biocompatibility, half-life and bioavailability as well as to control release of active pharmaceutical gradients from nanomedicines in body. In particular, Paxone^®^, Ulasta^®^, and PLEGRIDY^®^ formulated with the use of poly(ethylene glycol) (PEG) are representative polymer nanomedicines resulting in increased half-life and bioavailability in in vivo. Micelles include Estrasorb^®^, BIND-014, and CALAA-01 as controlled-release forms of lipophilic drugs. Liposomes have reduced toxicity and increased bioavailability, and include Onivyde^®^, Doxil^®^, Visudyne^®^, and Thermodox^®^. Antibody-drug conjugates (ADCs) have been used to reduce drug cytotoxicity and improve solubility (PEGylation). ADCs are stable in blood and within targeted cancer cells and are expected to be released into intracellular or paracellular compartments after uptake. The pairing and linkage of antibody and drug are important, and are critical factors for their slow clearance and long half-life (approximately 3 and 4 days). Brentuximab emtasine is an example of an ADC nanomedicine which addresses safety issues by reducing toxicity of monomethyl auristane E. In this case, maleimide linkage and conjugation with thiolated antibody results in the release of only 2% monomethyl auristane E even 10 days after administration. ADCs with non-cleavable linkages such as those with tratuzumab are also available. Nanomedicines using protein nanoparticles include Abraxane^®^, an albumin-bound paclitaxel, and Ontak^®^, an engineered fusion protein, which consist of endogenous or engineered protein carriers. Inorganic nanoparticles in nanomedicine are drug formulations commonly used for treatment and/or imaging, in which metallic and metal oxide materials are used. Coating with hydrophilic polymers (dextran or sucrose) such as iron oxide is used for iron supplements including Venofer^®^, Ferrlecit^®^, INFed^®^, Dexferrum^®^, and Feraheme^®^, which show slow dissolution patterns after intravenous administration and less toxicity due to free iron in high dosage regimens. Because poor absorption of free iron is one of the reasons for increasing iron dosage resulting in severe toxicity, an iron oxide nanomedicine formulation with iron supplementation is clinically meaningful. Inorganic nanomedicines using gold are based on thermal and surface chemistry of gold, and it have not yet been approved by the FDA. Several clinical investigations using nanomedicines formulated with gold have been conducted. CYP-6091 containing colloidal gold with recombinant human tumor necrosis factor rhTNF is in a phase 2 trial, NBTXR3 and PEP503 are radio enhancers containing hifnium metal oxide for brain tumor treatment and inorganic silica nanoparticles for fluorescence-based cancer imaging, respectively, and are in phase 1 trials. Nanocrystal formulations increase nanoscale dimensions and improve dissolution and solubility and include Rapamune^®^, Tricor^®^, Emend^®^, and Megace ES^®^.

**Table 4:** Nanomedicines approved by FDA

Formulations	Product names	Pharmaceutical company	Indications	Characteristics	Approval year
Polymer NP: synthetic polymer particles					
PEGylated adenosine deaminase enzyme	Adagen^®^/pegademase bovine	Sigma-Tau Pharmaceuticals	Serious immunodeficiency therapy	Improved circulation (retention) in body and decreased immunogenicity	1990
PEGylated antibody fragment (Certolizumab)	Cimzia^®^/certolizumabpegol	UCB	Chron’s disease, rheumatoid arthritis, psoriasis, ankylosing spondylitis	Improved circulation (retention) in body and stability	2008 2009 2013
Random copolymer of l-glutamate, l-alanine, l-lysine and l-tyrosine	Copaxone^®^/Glatopa	Teva	Multiple sclerosis	Regulation of CL by large amino-acid polymers	1996
Leuprolide acetate and polymer [PLGH(poly(dl-lactide-coglycolide)]	Eligard^®^	Tolmar	Prostate cancer	Regulation of drug delivery by prolongation of circulation (retention) in body	2002
PEGylated anti-VEGF aptamer (vascular endothelial growth factor) aptamer	Macugen^®^/Pegaptanib	Bausch&Lomb	Decreased vision	Improved aptamier stability by PEGylation	2004
Chemically synthesized ESA (erythropoiesis-stimulating agent)	Mircera^®^/Methoxy PEG glycol-epoetin β	Hoffman-LaRoche	Anemia with chronic renal failure	Improved aptamier stability by PEGylation	2007
PEGylated GCSF protein	Neulasta^®^/pegfilgrastim	Amgen	Leukopenia by chemotherapy	Improved protein stability by PEGylation	2002
PEGylated IFN alpha-2a protein	Pegasys^®^	Genentech	Hepatitis B and C	Improved protein stability by PEGylation	2002
PEGylated IFN alpha-2b protein	PegIntron^®^	Merck	Hepatitis C	Improved protein stability by PEGylation	2001
Poly(allylamine hydrochloride)	Renagel^®^ [sevelamer HCl]/Renagel^®^ [sevelamer carbonate]	Sanofi	Chronic renal failure	Regulation of drug delivery by prolongation of circulation (retention) in body and increased target delivery	2000
PEGylated HGH receptor antagonist	Somavert^®^/pegvisomant	Pfizer	Acromegaly	Improved protein stability by PEGylation	2003
Polymer-protein conjugate PEGylated l-asparaginase	Oncaspar^®^/pegaspargase	EnzonPharmaceuticals	Acute lymphocytic blood clot	Improved protein stability by PEGylation	1994
Polymer-protein conjugate (PEGylated porcine-likeuricase)	Krystexxa^®^/pegloticase	Horizon	Chronic gout	Improved protein stability by PEGylation	2010
Polymer-protein conjugate (PEGylated IFNbeta-1a)	Plegridy^®^	Biogen	Multiple sclerosis	Improved protein stability by PEGylation	2014
Polymer-protein conjugate (PEGylated factor VIII)	ADYNOVATE	Baxalta	Hemophilia	Improved protein stability by PEGylation	2015
Liposome					
Liposomal daunorubicin	DaunoXome^®^	Galen	Karposi sarcoma	Increased drug delivery to tumor cells and decreased systemic toxicity	1996
Liposomal cytarabine	DepoCyt©	Sigma-Tau	Lymphoma	Increased drug delivery to tumor cells and decreased systemic toxicity	1996
Liposomal vincristine	Marqibo^®^	Onco TCS	Acute lymphocytic blood clot	Increased drug delivery to tumor cells and decreased systemic toxicity	2012
Liposomal irinotecan	Onivyde^®^	Merrimack	Pancreatic cancer	Increased drug delivery to tumor cells and decreased systemic toxicity	2015
Liposomal amphotericin B	AmBisome^®^	Gilead Sciences	Fungal infection	Reduced renal toxicity	1997
Liposomal morphine sulphate	DepoDur^®^	Pacira Pharmaceuticals	Loss of pain due to surgery	Prolonged exposure	2004
Liposomal verteporfin	Visudyne^®^	Bauschand Lomb	Decreased vision, Ophthalmic hiscomaplastia	Improved drug delivery to lesion vessels and photosensitivity	2000
Liposomal doxorubicin	Doxil^®^/Caelyx™	Janssen	Karposi sarcoma, ovarian cancer, Multiple myeloma	Increased drug delivery to target sites and decreased systemic toxicity	1995 2005 2008
Liposomal amphotericinB lipid complex	Abelcet^®^	Sigma-tau	Fungal infection	Reduced toxicity	1995
Liposome-proteins SP-band SP-C	Curosurf^®^/Poractantalpha	Chieseifarmaceutici	Lung activator for stress disorder	Increased drug delivery at low dose and decreased toxicity	1999
Micelles					
Micellar estradiol	Estrasorb™	Novavax	Menopause hormone Therapy	Clinically release control	2003
Protein NP					
Albumin-bound paclitaxel NP	Abraxane^®^/ABI-007	Celgene	Breast cancer, non-small cell lung cancer, pancreatic cancer	Improved solubility and drug delivery to target tissues	2005 2012 2013
Engineered protein combining L-2 and diphtheria toxin	Ontak^®^	Eisai Inc	T-Cell lymphoma	T cell-selective targeting	1999
Nanocrystal					
Aprepitant	Emend^®^	Merck	Vomiting agent	Rapid absorption and increased F	2003
Fenofibrate	Tricor^®^	Lupin Atlantis	Hyperlipidemia	Increased F	2004
Sirolimus	Rapamune^®^	Wyeth Pharmaceuticals	Immunosupressant	Increased F and decreased dose	2000
Megestrol acetate	MegaceES^®^	Par Pharmaceuticals	Anorexia	Increased F and decreased dose	2001
Morphine sulfate	Avinza^®^	Pfizer	Mental stimulant	Increased F and decreased dose	2002 2015
Dexamethyl-phenidate HCl	Focalin XR^®^	Novartis	Mental stimulant	Increased F and decreased dose	2005
Metyhlphenidate HCl	Ritalin LA^®^	Novartis	Mental stimulant	Increased F and decreased dose	2002
Tizanidine HCl	Zanaflex^®^	Acorda	Muscle relaxant	Increased F and decreased dose	2002
Calcium phosphate	Vitoss^®^	Stryker	Bone substitute	Imitation of bone structure by cell adhesion and growth	2003
Hydroxyapatite	Ostim^®^	Heraseus Kulzer	Bone substitute	Imitation of bone structure by cell adhesion and growth	2004
Hydroxyapatite	OsSatura^®^	IsoTis Orthobiologics	Bone substitute	Imitation of bone structure by cell adhesion and growth	2003
Hydroxyapatite	NanOss^®^	Rti Surgical	Bone substitute	Imitation of bone structure by cell adhesion and growth	2005
Hydroxyapatite	EquivaBone^®^	Zimmer Biomet	Bone substitute	Imitation of bone structure by cell adhesion and growth	2009
Paliperidone Palmitate	Invega^®^Sustenna^®^	Janssen Pharms	Schizoaffective disorder	Control of slow release rate in drugs with low solubility	2009 2014
Dantrolene sodium	Ryanodex^®^	Eagle Pharmaceuticals	Malignant benign hypothermia	Rapid absorption at high dose	2014
Inorganic/metallic NPs					
Iron oxide	Nanotherm^®^	MagForce	Hybrid species	Vertical irritant effect by increased uptake	2010
Ferumoxytol SPION with poly glucose sorbitol carboxy methylether	Feraheme™/ferumoxytol	AMAG pharmaceuticals	Chronic renal failure with iron deficiency	Extended release and reduced dose	2009
Iron sucrose	Venofer^®^	Luitpold Pharmaceuticals	Chronic renal failure with iron deficiency	Increased dose capacity	2000
Sodium ferric gluconate	Ferrlecit^®^	Sanofi Avertis	Chronic renal failure with iron deficiency	Increased dose capacity	1999
Iron dextran (low MW)	INFeD^®^	Sanofi Avertis	Chronic renal failure with iron deficiency	Increased dose capacity	1995
Iron dextran (high MW)	DexIron^®^/Dexferrum^®^	Sanofi Avertis	Chronic renal failure with iron deficiency	Increased dose capacity	1997
SPION coated with dextran	Feridex^®^/Endorem^®^	AMAG pharmaceuticals	Imaging materials	Vertical irritant effect	1996 2008
SPION coated with dextran	GastroMARK™/umirem^®^	AMAG pharmaceuticals	Imaging materials	Vertical irritant effect	2001 2009

#### Suggested considerations for the evaluation of nanomedicines by the FDA

Based on guidelines and reports from the FDA, considerations for evaluation of nanomedicines are as follows. Evaluation of nano-formulation properties of nanomedicines comprises evaluating physicochemical properties of the nanomaterials, constituents and proportions of the nanomaterials, and quality and manufacturing of the nanomaterials (Eifler and Thaxton 2011; FDA 2010). First, pharmacokinetics of nanomedicines are assessed in the context of their systemic exposure considering (1) rate and amount of absorption and retention in circulation based on blood concentration over time, (2) relationship between prolongation of half-life and whole body exposure duration, and (3) bioavailability changes (Eifler and Thaxton 2011; FDA 2010, 2015). Second, assessment of nanomedicine distribution to blood and tissue is recommended to be done based on apparent volume of distribution, and distribution or accumulation to positive targeting sites based on time-dependent changes. Third, in the context of metabolism, it is important to evaluate whether decomposition or metabolism of nano-formulations or their active pharmaceutical ingredients occur. Fourth, elimination of raw materials used in nano-formulations, and products from decomposition and/or metabolism of nano-formulations and their active pharmaceutical ingredients are recommended for evaluation. The accumulation of nano-formulations in target tissues and elimination through MPS are also investigated. Finally, toxicity assessment of nanomedicines needs to be conducted.

### EMA

In 2011, the EMA defined nanomedicines as drugs composed of nanomaterials 1–100 nm in size, and these are classified into liposomes, nanoparticles, magnetic NPs, gold NPs, quantum dots, dendrimers, polymeric micelles, viral and non-viral vectors, carbon nanotubes, and fullerenes (EFSA 2011; EMA 2015a).

#### Status of nanomedicines approved by the EMA

The EMA has approved 8 of the 11 commercially available nanomedicine drugs developed as first-generation nanomedicines (such as liposomes or iron-containing formulations), and three of them were withdrawn. Investigations were conducted to establish the scientific basis for efficacy and safety of 12 nanomedicines, and were evaluated via the European Medicines Agency (EMA) approval process. Following this initial process, 48 nano medicines or imaging materials are currently in clinical trials (Phase 1–Phase 3) in the EU. In addition, preclinical trials are underway for a number of nanomedicine products (Draca et al. 2013; Ehmann et al. 2013; Hafner et al. 2014; Lawrence and Rees 2000; Ling et al. 2013; Shegokar and Müller 2010) (Table 5).

**Table 5:** Nanomedicines approved by EMA

Formulations	API	Product name	Pharmaceutical company	Administration route	Indications
Nanocrystals	Aprepitan	Emend^®^	Merck Sharp and Dohme BV	Capsule	Vomiting after surgery
	Fenofibrate	Tricor^®^/Lipanthyl^®^/Lipidil^®^	Recipharm, FR	Tablet	Hyperlipidemia
	Olanzapine	Zypadhera^®^	Lilly Pharma	Powder/solvent	Schizophrenia
	Paliperidone	Xeplion^®^	Janssen Pharmaceutica NV	Prolonged release suspension for injection (im)	Schizophrenia
	Sirolimus	Rapamune^®^	Pfizer Ireland Pharmaceuticals, IE	Tablet	Kidney transplantation rejection
Nanoemulsions	Cyclosporine	Norvir^®^	Aesica Queenborough Ltd	Soft capsules	HIV infection, kidney transplantation rejection
	Pegaspargase (mPEG-asparaginase)	Oncaspar^®^	Sigma-tau Arzneimittel GmbH	Solution (iv/im)	Acute lymphocytic leukemia
	Sevelamer	Renagel^®^/Renvela^®^	Genzyme Ltd	Tablet	Dialysis, hyperphosphatemia
Polymer-protein conjugates	Amphotericin B	AmBisome^®^	Gilead Sciences	Suspension (iv)	Fungal infection
	Certolizumabpegol (PEG-anti-TNFFab)	Cimzia™	UCB Pharma SA	Solution (sc)	Rheumatoid arthritis
	Methoxypolyethylene glycol-epoetin beta	Mircera^®^	Roche Pharma	Solution (iv/sc)	Anemia, chronic renal failure
	Pegfilgrastim (PEG-rhGCSF)	Neulasta^®^	Amgen Technology	Solution (sc)	Leukopenia by chemotherapy
	Peginterferonalpha-2a (mPEG-interferon alpha-2a)	Pegasys^®^	Roche Pharma	Solution (sc)	HBV/HCV infection
	Peginterferonalpha-2b (mPEG-interferon alpha-2b)	PegIntron^®^	Schering-Plough	Solution for injection (sc)	HIV inflammation
	Pegvisomant (PEG-HGH antagonist)	Somavert^®^	Pfizer Manufacturing	Solution for injection (sc)	Peripheral hypertrophy
Liposomes	Cytarabine	DepoCyt^®^	Almac Pharma	Suspension (intrathecal)	Brain cancer
	Daunorubicin	DaunoXome^®^	Gilead Sciences Ltd	Suspension (iv)	Kaposi sarcoma by HIV
	Doxorubicin	Myocet^®^	GP-Pharm	Suspension (iv)	Breast cancer
	Doxorubicin	Caelyx^®^	Janssen Pharmaceutical	Suspension (iv)	Breast cancer, ovarian cancer, Kaposi sarcoma
	Mifamurtide	Mepact^®^	Takeda	Suspension (iv)	Myosarcoma
	Morphine	DepoDur^®^	Almac Pharma	Suspension(epidural)	Pain
	Paclitaxel	Abraxane^®^	Celgene	Powder for suspension	Breast cancer
	Propofol	Diprivan^®^/Propofol-Lipuro^®^/Propofol^®^	Astra Zeneca	Emulsion (iv)	Anesthesia
	Verteporfin	Visudyne^®^	Novartis Pharma GmbH, Nürnberg	Suspension (iv)	Decreased vision, myopia
Nanoparticles	Inactivated hepatitis A virus	Epaxal^®^	Crucell	Suspension (iv)	Hepatitis A vaccines
	90Y-ibritumomab tiuxetan	Zevalin^®^	Bayer Pharma	Solution (iv)	Lymphoma
Virosomes	Adjuvanted influenza vaccine	Inflexal^®^ V	Crucell	Suspension (iv)	Influenza vaccines
	Glatiramer (Glu,Ala,Tyr,Lys copolymer)	Copaxone^®^	Teva Pharmaceuticals	Solution (sc)	Multiple sclerosis
Polymeric drugs	Sodium ferric gluconate	Ferrlecit^®^	Aventis Pharma	Solution (iv)	Anemia with iron deficiency
Nanocomplex	Ferric carboxymaltose	Ferinject^®^	Vifor	Solution (iv)	Iron deficiency
	Ferumoxytol	Rienso^®^	Takeda	Solution (iv)	Anemia with iron deficiency, chronic renal failure
	Iron sucrose [iron(III)-hydroxidesucrose complex]	Visudyne^®^	Novartis	Solution (iv)	Iron deficiency
	Iron(III) isomaltoside	Monofer^®^	Pharmacosmos	Solution (iv)	Iron deficiency
	Iron(III)-hydroxide dextran complex	Ferrisat^®^/Cosmofer^®^	Pharmacosmos	Solution (iv)	Iron deficiency

#### Suggesting points for the evaluation of nanomedicines in EMA

EMA presents that pharmacokinetic and pharmacodynamic properties of nanomedicines were determined by chemical composition and physicochemical properties. So, EMA suggest to consider six possibilities to evaluate nanomedicines considering the chemical composition and physicochemical properties (EFSA 2011; TGA 2016) including (1) nano-formulations are unstable at the time of manufacture and are converted into non-nanosized form, (2) the state of conversion into non-nanosized form when the drug substance in the manufacturing site is present as a matrix, (3) conversion to non-nanosized forms due to lack of bio-similarity under in vitro non-stable conditions, (4) conversion from nano-forms to non-nanosized forms during toxicity assessment (5) co-existence of nano forms and non-nano forms at the in vivo administration site, and (6) existence of the nano form in biological samples and tissues after absorption. In view of these various considerations for nanomedicine evaluation, EMA suggested the need to discuss the following aspects for the evaluation of nanomedicines (EFSA 2011; EMA 2015a, b; Ehmann et al. 2013; TGA 2016). Overall, physicochemical properties, stability, and functionality of nanomedicines should be evaluated. To this end, interactions and reactivity with biointerfaces due to coatings or additives in the final nanomedicines, suitability of biomarkers of in vivo functionality of nanomedicines, in vivo distribution and bio-persistence of nanomedicines, long-term safety of decomposition products, and adequacy of dose and dose interval settings have emerged as key factors for the evaluation process. Notably, liposome formulations, iron-based formulations, and nanocrystal formulations which can be considered first-generation nanomedicines and have already been marketed and used, have proved their effectiveness and safety over a long period. Based on this status, evaluation methods for approval of second-generation nanomedicines have been suggested for consideration (Ehmann et al. 2013; EMA 2013a, b, EMA 2015b).

### Future perspectives on nanomedicines considering their pharmacokinetic properties

**Fig. 1:**
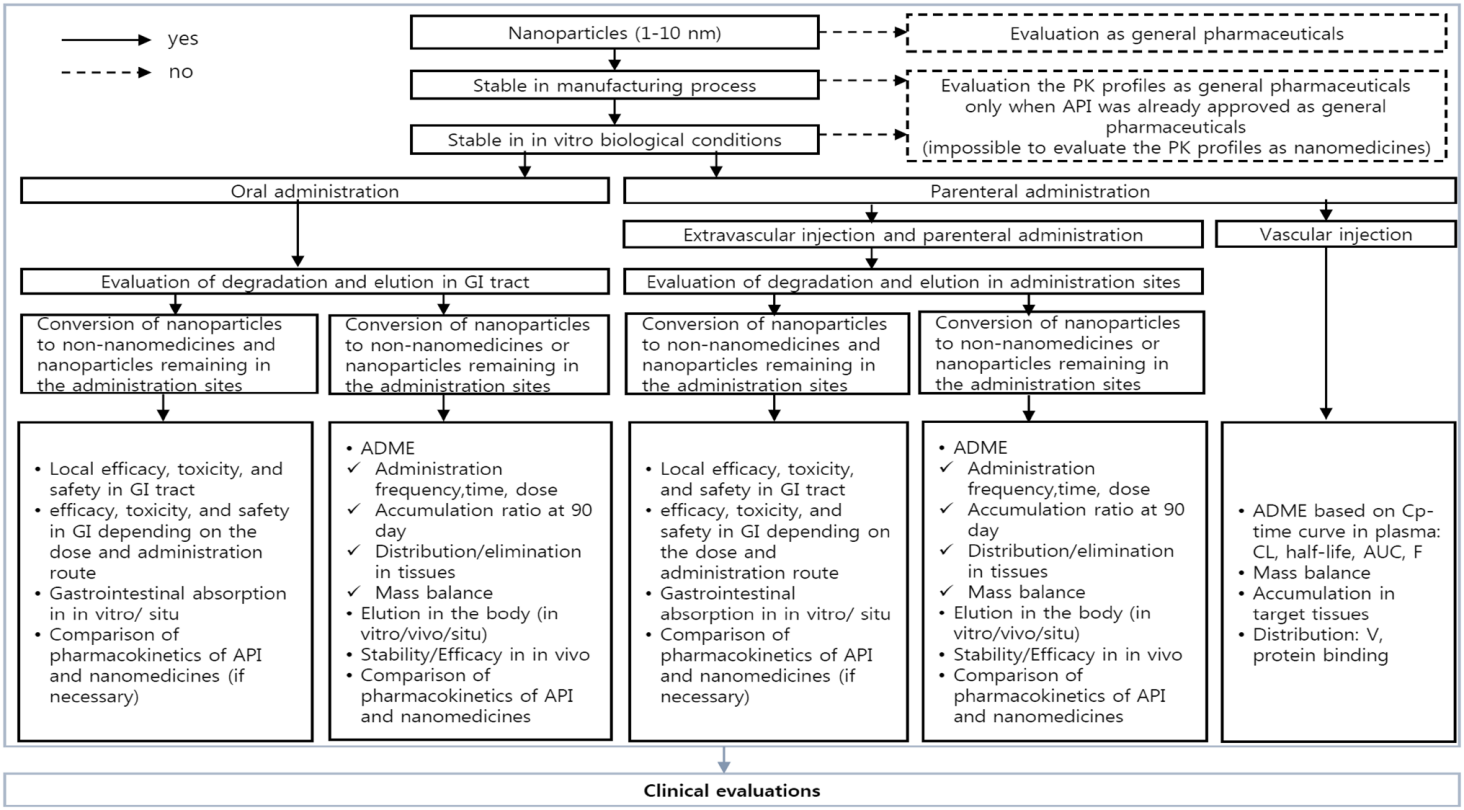
A proposed new algorithm to assess ADME of nanomedicines

Given the considerations for development and use of nanomedicines, indispensable steps to attain clinical significance include assessment of the nature of formulations, pharmacokinetic properties, and the approval process for nanomedicines. Therefore, based on recent trends in nanomedicine development and guidelines of the FDA and EMA, we propose a simple algorithm to guide the recommended ADME evaluations of nanomedicines (Fig. 1). In the proposed algorithm, stability in the manufacturing process and simulated human conditions determine whether ADME properties of the drugs of interest are assessed or not. Assessment varies based on administration routes and distribution. For example, evaluation varies based on whether orally administered nanomedicines are found in nano forms or non-nano forms in the gastro-intestinal tract. Thus, the proposed algorithm provides critical and practical checkpoints in nanomedicine development and assessment.
